# COPD and T2DM: a Mendelian randomization study

**DOI:** 10.3389/fendo.2024.1302641

**Published:** 2024-02-08

**Authors:** Tao Wang, Jinshuai Li, Chun Huang, Xiangjian Wu, Xiaoyan Fu, Chunfeng Yang, Minfang Li, Sheng Chen

**Affiliations:** The Fourth Clinical Medical College, Guangzhou University of Chinese Medicine, Shenzhen, China

**Keywords:** type 2 diabetes mellitus, Chronic Obstructive Pulmonary Disease, Mendelian randomization (MR) analysis, GWAS data, causal relationship

## Abstract

**Introduction:**

Type 2 diabetes (T2DM) stands as a global chronic illness, exerting a profound impact on health due to its complications and generating a significant economic burden. Recently, observational studies have pointed toward a potential link between Chronic Obstructive Pulmonary Disease (COPD) and T2DM. To elucidate this causal connection, we employed the Mendelian randomization analysis.

**Method:**

Our study involved a two-sample Mendelian randomization (MR) analysis on COPD and T2DM. Additionally, tests for heterogeneity and horizontal pleiotropy were performed.

**Results:**

For the MR analysis, 26 independent single nucleotides polymorphisms (SNPs) with strong associations to COPD were chosen as instrumental variables. Our findings suggest a pronounced causal relationship between COPD and T2DM. Specifically, COPD emerges as a risk factor for T2DM, with an odds ratio (OR) of 1.06 and a 95% confidence interval ranging from 1.01 to 1.11 (P = 0.006). Notably, all results were devoid of any heterogeneity or pleiotropy.

**Conclusion:**

The MR analysis underscores a significant causal relationship between COPD and T2DM, highlighting COPD as a prominent risk factor for T2DM.

## 1 Introduction

Over the past three decades, the prevalence of Type 2 diabetes (T2DM) and its associated complications have surged globally, especially in low- and middle-income nations. This expansion represents a burgeoning crisis that poses severe threats to both global health and economic prospects. It is estimated that over 9% of the global adult population is diagnosed with T2DM. Those affected by T2DM face an average reduction in life expectancy of 8 years in the US, accompanied by a myriad of complications that compromise their quality of life. Significantly, nearly 12% of the world’s health expenditures in 2015 were dedicated to addressing T2DM and its consequential complications (1). The etiological factors underlying T2DM are diverse, intertwining genetic, epigenetic, and lifestyle determinants within an expansive physical-socio-cultural milieu.

Chronic Obstructive Pulmonary Disease (COPD) represents a significant global health challenge. As of 2015, an estimated 7.3 billion individuals worldwide were affected by COPD. With an aging population and persistent smoking habits, the prevalence of such chronic diseases is expected to rise (2). Notably, numerous studies indicate a higher incidence of diabetes in COPD patients (3–6). For instance, a retrospective investigation led by Mario Cazzola, which encompassed 341,329 Italian participants from the Health Search Database (HSD), discerned that individuals without COPD bore a significantly reduced prevalence of diabetes than their COPD-afflicted counterparts. However, the occurrence of metabolic syndrome remained stable irrespective of COPD, underscoring the hypothesis that COPD, rather than metabolic syndrome, amplifies the risk of diabetes (6). This pattern suggests a potential pathophysiological intertwining between COPD and T2DM.

A recent systematic review underscored that COPD could be a harbinger for the advancement of diabetes, emphasizing that individuals with severe to very severe COPD face a heightened risk of diabetes onset (7, 8). However, a prospective analysis reported no discernible link between obstructive pulmonary function impairment and T2DM (9). Even though the systematic review probed the potential nexus between COPD and diabetes, its conclusions remain inconclusive. Many investigations centering on COPD encompass a restricted cohort, and subjects diagnosed with AECOPD/T2DM frequently exhibit other pronounced co-morbidities, clouding the interpretation of metrics such as hospital stay durations and mortality rates. Furthermore, the perceived interrelation between COPD and diabetes could be a byproduct of external influencers like smoking (10), as opposed to intrinsic pathophysiological ties (8). Such nuances introduce biases when scrutinizing outcomes tethered to both ailments, obfuscating the delineation of a direct linkage between COPD and T2DM.

Mendelian Randomization (MR) is an innovative analytical tool that employs genetic variables to elucidate causal dynamics between exposures and outcomes (11, 12). By harnessing the association between genetic variants and specific modifiable exposures, MR affords insights into the causal ramifications of such exposures. Given the inherent randomness of genetic inheritance during conception, MR stands insulated from external confounders. Furthermore, the immutable nature of genetic variations, fixed at conception, shields the analysis from subsequent health or lifestyle influences (13). In contrast to conventional observational studies, MR offers a fortified stance against typical confounders and biases, grounding causal inferences in a more solid bedrock. Due to the predetermination of genetic variations, MR sidesteps pitfalls like reverse causation. It also leverages these variations as surrogates for prolonged exposures, refining assessments and countering the errors and biases typical of observational research (14). Under the appropriate conditions, MR can be harnessed in expansive research frameworks to probe if COPD indeed acts as a precursor to T2DM.

In our research, we employed Mendelian randomization analyses on separate samples for COPD and T2DM with the goal of elucidating a causal link between these two disorders. If such a causal association is substantiated, strategies aimed at treating COPD could potentially be leveraged to mitigate the onset and progression of T2DM.

## 2 Method

### 2.1 Exposure populations

For our COPD susceptibility investigation, we analyzed data from 257,811 individuals spanning 25 studies, all of European ancestry. We defined COPD specifically using prebronchodilator spirometry criteria: a Forced Expiratory Volume in the first second (FEV1) less than 80% of the predicted value, along with a FEV1 to Forced Vital Capacity (FVC) ratio under 0.7., we pinpointed 82 SNPs, distinguishing 35,735 COPD cases from 222,076 controls. This GWAS took intoaccount age, age^2^, gender, height, principal components, and smoking habits (14).

### 2.2 Outcome populations

**Figure 1:**
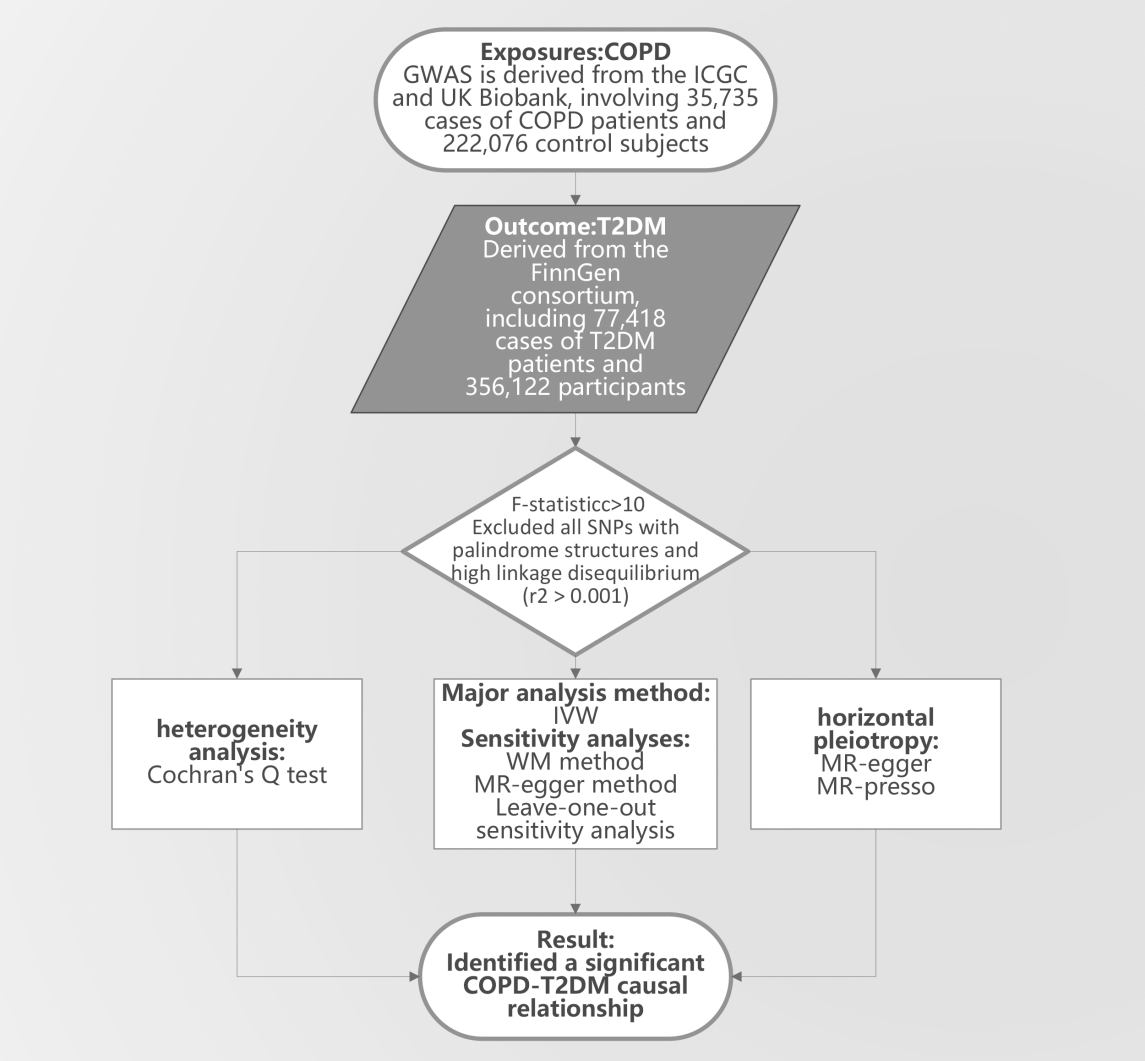
The study design. COPD, Chronic Obstructive Pulmonary Disease; T2DM, Type 2 diabetes mellitus; GWAS, genome-wide association study; IVW, Inverse Variance Weighting; MR-egger, Mendelian Randomization-Egger Regression; WM, Weighted Median; MR-presso, Medelian Randomization Pleiotropy RESIdual Sum and Outlier.

In our research on T2DM, we utilized GWAS summary data from Japan, corresponding to IEU identifier: ebi-a-GCST010118, encompassing 433,540 individuals predominantly from East Asian backgrounds. To define the T2DM case-control, each included study adhered to at least one or more of these established criteria (1): A confirmed doctor’s diagnosis or ongoing diabetes medication regimen (2), fasting blood glucose levels of ≥126mg/dL (3), fasting blood glucose levels exceeding 200mg/dL (4), random blood glucose readings of ≥200mg/dL, or (5) HbA1c levels of ≥6.5%. Following these guidelines, our sample consisted of 77,418 T2DM diagnosed patients and 356,122 control subjects. It’s noteworthy that the GWAS factored in adjustments for age, gender, and body mass index [16]. The research design is shown in **Figure 1**. For further details on the GAWS phenotype definitions related to COPD and T2DM, as well as baseline data, please refer to the **Supplementary Material** (15).

### 2.3 Mendelian Randomization analysis

Mendelian Randomization (MR) studies are grounded in three core assumptions: a. The genetic variants utilized as Instrumental Variables (IVs) should have a robust association with the targeted risk factors. b. These IVs should not correlate with other potential confounders affecting the link between risk factors and outcomes. c. The connection between the IV and the outcomes should be entirely mediated through the risk factors (13).

With these principles as our foundation, we cherry-picked SNPs showing a significant correlation to ‘COPD’ (p < 5 × 10^-8) to serve as our IVs. We gauged the strength of the link between these IVs and exposure by computing their F-statistics (F-statistics = β_2_/SE_2_). SNPs presenting f-statistics <10 were discarded since larger f- statistics suggest the genetic markers cause phenotypic variations, with ensuing results diverging due to these variations (16). We then underwent LD-clumping (r^2^ > 0.001) and sidestepped all palindromic SNPs (17–19). In our broad-spectrum ensitivity scrutiny, we vetted the remaining SNPs on the PhenoScanner portal and filtered out those linked with cardiovascular ailments, hypertension, and hyperlipidemia (20, 21). The MR-presso method facilitated our test for horizontal pleiotropy, allowing us to eject outliers (22). When MR pinpointed SNPs inherently tied to exposure in the findings, we inferred a causal influence of the exposure on the results.

Our main analytic tool was the Inverse Variance Weighting (IVW) method, which synthesized SNP causal impacts, counterbalanced by the ratio estimate variances (19, 23). The MR-egger regression’s slope rendered authentic causal parameters (24). Complementarily, the Weighted Median technique, assuming the validity of 50% of the IVs, fine-tuned the reliability of causal interpretations, curbing Type I errors (25).

We employed the MR-egger intercept to gauge horizontal pleiotropy, spotting its presence if the intercept deviated from zero (26). In scenarios sans horizontal pleiotropy, the principal causal effect was drawn from the IVW method’s random effects. Cochran’s Q test came into play to discern heterogeneity, which, if present, contravenes the IV tenet (23). To reinforce our findings’ solidity, we implemented a eave-one-out sensitivity analysis, sequentially eliminating each SNP to pinpoint anomalies.

In this study, we utilized Odds Ratios (OR) and 95% confidence intervals to elineate the causal relationship between exposure and outcome. We considered a causal relationship as established when it was consistently evidenced across both heterogeneity tests and sensitivity analyses. All statistical analyses were conducted using R version 4.3.1 in R Studio, with a primary reliance on the TwoSampleMR (version 0.5.7) and MR-PRESSO packages (version 1.0.0) for the two-sample MR analysis.

## 3 Results

**Figure 2:**
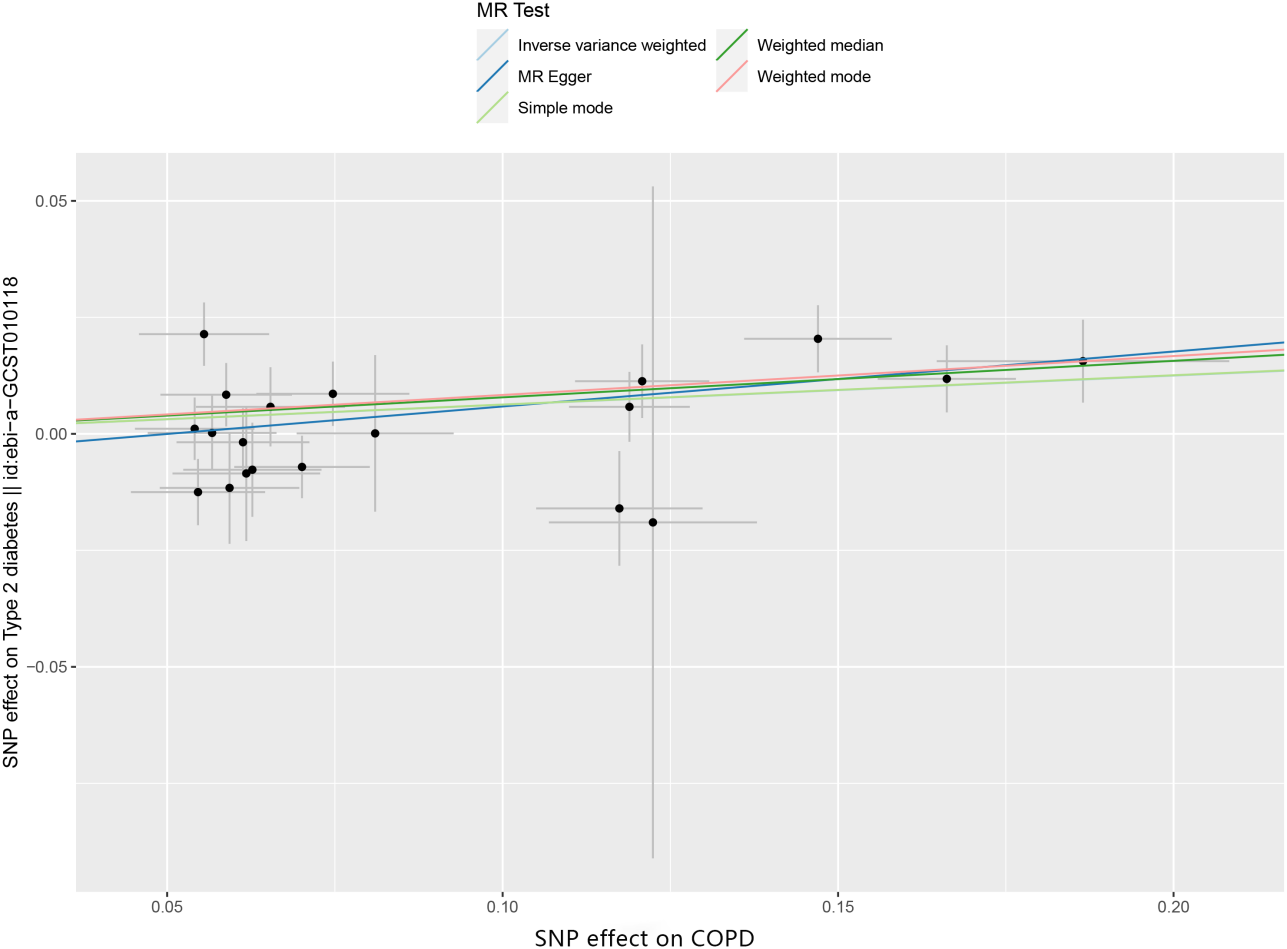
Scatter plot depicting the casual effevt of COPD on the odds ratio for T2DM.

We identified 26 independent SNPs with a robust association with T2DM, serving as instrumental variables. All exhibited an f-statistic exceeding 10, effectively ruling out weak instrument bias. Interestingly, the MR-Egger intercept closely aligned with 0 (p=0.263), suggesting no evidence of horizontal pleiotropy in our analysis (as illustrated in **Figure 2**). On the other hand, the initial MR-PRESSO global test returned a P-value below 0.05. Upon exclusion of three outlier SNPs — namely “rs4888379”, “rs7068966”, and “rs76841360” — the subsequent MR-PRESSO global test yielded a p-value exceeding 0.05. This result, post the outlier exclusion, supports the absence of horizontal pleiotropy in the MR-PRESSO findings. Furthermore, Cochran’s Q test detected no heterogeneity (p>0.05, Q=27.080). Given this lack of heterogeneity, there was no notable precision disparity between the random and fixed effects models, prompting our choice of the IVW random effects model for our analyses (27).

**Figure 3:**
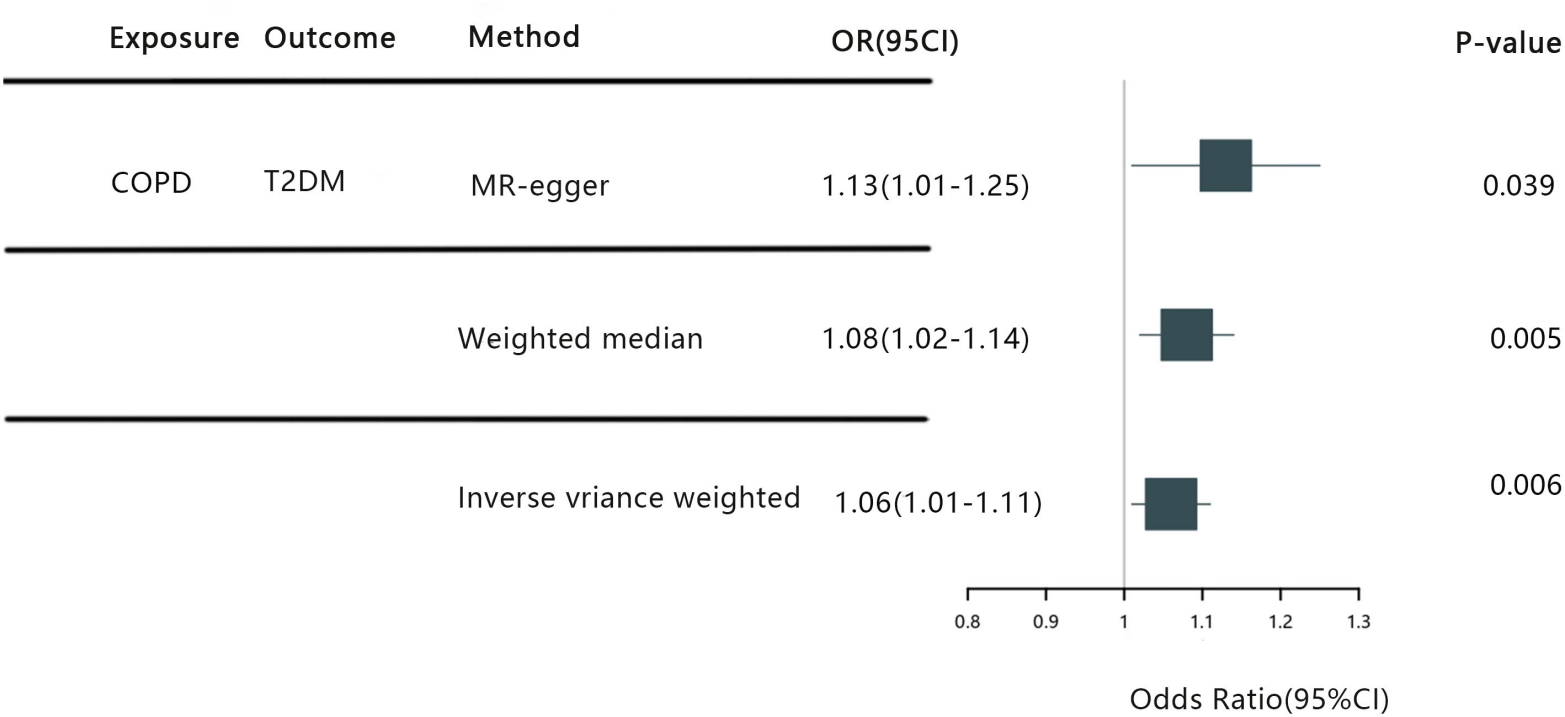
COPD, Chronic Obstructive Pulmonary Disease; T2DM, Type 2 diabetes mellitus; IVW, Inverse Variance Weighting; MR-egger, Mendelian Ra.

The IVW random effects model returned an OR of 1.06 (95%CI: 1.01-1.11, P=0.006), while the MR-Egger regression reported an OR of 1.13 (95%CI: 1.01-1.25, p=0.039). Additionally, the weighted median test registered an OR of 1.08 (95%CI: 1.02-1.14, p=0.005) (See **Figure 3**). Conclusively, the trio of IVW, MR-Egger, and weighted median methods all reinforce a causal link between COPD and T2DM, firmly positing COPD as a predisposing factor for T2DM. In particular, our IVW findings suggest that individuals with COPD are faced with a heightened risk of T2DM by approximately 6.5% in comparison to those without COPD. Importantly, the leave-one-out sensitivity analysis underscored the consistency and reliability of our findings, establishing that the omission of any single SNP failed to considerably sway our overarching results.

## 4 Discussion

COPD and T2DM, both chronic diseases of global concern, have been the focus of numerous studies due to their widespread prevalence. While many observational studies consistently report an elevated incidence of T2DM among COPD patients (3–6), our findings provide pivotal evidence of a causal link, positioning COPD as a significant risk factor for T2DM. Significantly, our research stands out as the inaugural MR study delving into the causal influence of COPD on T2DM. Our conclusions resonate with the outcomes of several prior studies that also hint at the possible link between COPD and the emergence and progression of T2DM. For instance, a retrospective analysis by Chao-Shsun Lin et al. from Taiwan brought to light the augmented risk of T2DM in COPD patients, even in the absence of acute exacerbations, relative to their non-COPD counterparts. In line with this, T2DM patients with antecedent COPD had heightened ICU admissions, pneumonia instances, and mortality rates (28). In another enlightening study from Taiwan, Charles T.-C. Lee et al. affirmed that, even after accounting for potential confounders like hypertension, coronary heart disease, and age among others, COPD persisted as a standalone risk factor for T2DM (4). Corroborating this trend, Birgitte F’s nationwide observational research from Denmark pinpointed a risk increment of diabetes in COPD-afflicted individuals, which was approximately 20% higher than in those without COPD (3).

While some research posits a lack of significant correlation between COPD and diabetes onset, it’s imperative to acknowledge the association of COPD with a heightened diabetes risk (risk ratio = 1.45, 95% CI 1.04-2.03) (29). An illustrative example comes from a population-based survey by Hyejin Joo et al. from Korea, which found no distinct association between COPD and diabetes (30). Nevertheless, a caveat remains: many such studies are region-specific cross-sectional investigations, potentially reducing their global applicability. A critical challenge in these studies lies in the ambiguous diagnosis criteria for COPD, with a heavy reliance on either self- reported data or physician diagnoses, thereby obscuring the causal nexus. The lacunae in these findings accentuate the need for more compelling evidence to establish the COPD-T2DM causality.

Mendelian randomization (MR) has emerged as a potent statistical approach, harnessing genetic variances as instrumental variables to derive causal inferences. Capitalizing on the inherent randomness of genetic allocation, MR offers more causation-aligned estimates, enhancing the evidence’s robustness. A distinct edge MR holds is its ability to treat genetic variations as inherent randomized trials, side- stepping ethical or feasibility constraints typically encountered in traditional research paradigms. Amplifying its potency, MR adeptly taps into vast genetic data pools and GWAS findings, facilitating refined causal conclusions (13). Embarking on this backdrop, our investigation pioneers the MR exploration into the COPD-T2DM dynamics. Predominantly, we employed the IVW random effects model for causal delineation, unearthing a marked causal linkage between COPD and T2DM.

Complementing this, we integrated MR-Egger regression’s slope estimation, the weighted median method, and a rigorous leave-one-out sensitivity assessment. Concordantly, all methodologies reinforced the IVW’s conclusions. Notably, our assessment remained unmarred by pleiotropy, as evidenced by both MR-Egger intercept and MR-PRESSO global tests, and the Cochran’s Q test underscored a heterogeneity-free landscape. Collectively, these rigorous assessments underpin our conviction regarding the causal interplay between COPD and T2DM presented in this study.

The association between COPD and T2DM can be understood through a multifaceted lens. It’s well-documented that genetic predispositions significantly influence the susceptibility to T2DM: individuals with a T2DM-afflicted parent face a 40% lifetime risk, which amplifies to 70% when both parents are affected (31). Corroborating this genetic interplay, a comprehensive cohort study from Denmark unveiled a correlation coefficient of 0.43 between COPD and T2DM, hinting at intertwined genetic underpinnings of the two conditions (32). Such findings bolster the hypothesis that the co-occurrence of COPD and T2DM may stem from mutual genetic factors.

Inflammation, especially in the innate immune system, is a key link between COPD and T2DM (33, 34). The NLRP3 inflammasome, central to this process, releases cytokines like IL-1β and IL-18, which are important in COPD (33) and disrupt insulin signaling in T2DM. Additionally, the gut microbiota’s interaction with the NLRP3 inflammasome influences insulin sensitivity and signaling (34). Oxidative stress is a key factor in both COPD and T2DM (35). In T2DM, elevated reactive oxygen species (ROS) levels decrease insulin sensitivity, leading to insulin resistance. This affects the β-cells in the islets, reducing insulin secretion. Oxidative stress impairs mitochondrial ATP production and activates stress pathways, disrupting insulin signaling (36). In COPD, patients exhibit autoantibodies against carbonyl-modified self-proteins due to oxidative stress, causing lung tissue damage. Oxidative stress also attracts immune cells, increasing IL-17 and IL-18 levels and contributing to lung injury (37). We speculate that oxidative stress may contribute to insulin resistance in patients with COPD (38).

Diving into the daily lives of COPD patients, sedentary habits, like extended, sitting or lying durations, might pave the way for muscle-atrophying obesity, a precursor for T2DM (39–41). Obesity, unequivocally, is a heavyweight risk factor for T2DM (42). On another note, the medications for COPD might also play a part. Specifically, the use of inhaled corticosteroids (ICS) — a staple in COPD management— has been tied to T2DM onset (43). Mechanistically, glucocorticoids might sabotage.β-cell function and amplify insulin resistance (44), providing a rationale for why COPD might accentuate T2DM risk.

To incorporate the research findings into public health campaigns, the focus should be on educating about the COPD-T2DM link, advocating for regular check-ups and lifestyle changes for COPD patients, and stressing the importance of early intervention for T2DM prevention and management. This approach can also inform healthcare policies for better COPD patient care, considering their increased T2DM risk.

In this pioneering study, we leveraged two extensive GWAS datasets to establish, for the first time, a causal link between COPD and T2DM. This discovery has significant implications for the treatment and prevention of T2DM. Public health initiatives should incorporate these findings, focusing on educating about the COPD-T2DM connection and advocating for regular health check-ups and lifestyle changes in COPD patients. These measures are crucial for early T2DM intervention. Moreover, this insight can influence healthcare policies to improve care for COPD patients, given their increased risk of T2DM. Additionally, our study emphasizes the importance of a careful risk-to- benefit assessment when prescribing corticosteroids. We minimized biases by selecting European cohorts for COPD exposure and Asian cohorts for T2DM outcomes. Our methodological rigor, evident in the selection of instrumental variables with high F- statistics, strengthens the reliability of our findings. However, the study has limitations, such as unaddressed confounders like dietary habits and drug use, and it doesn’t explore the specific biological mechanisms connecting COPD to T2DM. Future research should focus on these aspects, using molecular, genetic, and more detailed longitudinal studies tracking COPD medication use and diabetes onset. to further elucidate this relationship.

## 5 Conclusion

In conclusion, our article stands as the pioneering work utilizing the MR method to probe the causal relationship between COPD and T2DM. We not only identified but also quantified the causal effect of COPD on T2DM. Our findings offer valuable insights for clinicians, guiding the management, treatment, and prevention strategies for patients co-diagnosed with COPD and T2DM. Moreover, this research lays a foundational bedrock for further investigations into the underlying mechanisms linking COPD and T2DM.

## Data availability statement

The original contributions presented in the study are included in the article/**Supplementary Material**. Further inquiries can be directed to the corresponding author.

## Ethics statement

Ethical approval was not required for the study involving humans in accordance with the local legislation and institutional requirements. Written informed consent to participate in this study was not required from the participants or the participants’ legal guardians/next of kin in accordance with the national legislation and the institutional requirements.

## Author contributions

TW: Conceptualization, Data curation, Formal analysis, Investigation, Methodology, Project administration, Resources, Software, Supervision, Validation, Visualization, Writing – original draft, Writing – review & editing. JL: Conceptualization, Data curation, Investigation, Methodology, Software, Supervision, Visualization, Writing – original draft, Writing – review & editing. CH: Conceptualization, Data curation, Investigation, Methodology, Software, Writing – review & editing. XW: Conceptualization, Methodology, Project administration, Validation, Writing – review & editing. XF: Methodology, Software, Supervision, Writing – review & editing. CY: Conceptualization, Data curation, Methodology, Supervision, Validation, Writing – review & editing. ML: Conceptualization, Data curation, Writing – review & editing. SC: Conceptualization, Funding acquisition, Investigation, Methodology, Resources, Software, Supervision, Validation, Writing – review & editing.
