# Supplementary material for: COPD and T2DM: a Mendelian randomization study
Source: Front Endocrinol (Lausanne). 2024 Feb 8;15:1302641. doi: 10.3389/fendo.2024.1302641 (PMC10883379; doi:10.3389/fendo.2024.1302641)
Supplement: Supplementary file 1 [file DataSheet_1.docx]

Supplementary Material

# Supplementary Data

# COPD and T2DM: A Mendelian Randomization Study

Tao Wang Chun Huang Jinshuai LI Xiangjian Wu Xiaoyan Fu Chunfeng Yang Chen Sheng

Contents

Table S1 Definition of GWAS phenotype for each disease.

Table S2 Baseline data of GWAS phenotype for each disease.

Table S3 Valid instrumental variables for Mendelian randomization analysis of COPD (exposure) on T2DM (outcome).( See Supplementary Table 2)

Table S4 MR-PRESSO test of the instrumental variables for COPD on T2DM.

Table S5 Heterogeneity test of the instrumental variables for COPD on T2DM. ( See Supplementary Table 2)

Table S6 Pleiotropy test of the instrumental variables for COPD on T2DM. ( See Supplementary Table 2)

Table S7 Inverse variance weighted、MR Egger、Weighted median test of the instrumental variables for COPD on T2DM. ( See Supplementary Table 2)

Figure 1: Flowchart for MR Analysis of COPD and T2DM

Figure 2: Forest Plot of the Main Results from the MR Analysis of COPD and T2DM

Figure S3 Scatter plot of the instrumental variables for COPD on T2DM.

Figure S4 MR leave-one-out sensitivity analysis for COPD on T2DM

Figure S5 Funnel plot for COPD on T2DM

**Table S1. Definition of GWAS phenotype for each disease.**

| **Trait** | **GWAS Phenotype Definition** |
| --- | --- |
| COPD | COPD was defined based on the modified Global Chronic Obstructive Pulmonary Disease Initiative (GOLD) criteria (moderate to very severe airflow limitation) and pre-bronchodilator spirometry (FEV1/FVC<0.7, FEV1% predicted<80%) |
| T2DM | For the case-control definition of T2DM, each study met one or more of the following criteria: (1) diagnosed by a physician and/or currently receiving diabetes medication, (2) fasting blood glucose ≥126mg/dL, (3) fasting blood glucose ≥200mg/dL, (4) random blood glucose ≥200mg/dL, or (5) HbA1c≥6.5%. Based on this definition |

COPD:chronic obstructive airways disease; T2DM: Type 2 diabetes mellitus ;GWAS, genome-wide association studies.

**Table S2 Baseline data of GWAS phenotype for each disease.**

|  | **cases** | **noncases** | **Male** | **Population** |
| --- | --- | --- | --- | --- |
| COPD | 35,735 | 222,076 | NA | European |
| T2DM | 54,481 | 224,231 | NA | East Asian |

COPD:chronic obstructive airways disease; T2DM: Type 2 diabetes mellitus ;GWAS, genome-wide association studies.

**Table S4 MR-PRESSO test of the instrumental variables for COPD on T2DM**

| **Exposure** | **Outcome** | **MR-PRESSO global Test** | ***p***  **(global test)** | ***p***  **(distortion test)** |
| --- | --- | --- | --- | --- |
| COPD | T2DM | 29.49 | 0.17 | NA |

COPD:chronic obstructive airways disease; T2DM: Type 2 diabetes mellitus ;GWAS, genome-wide association studies.MR-PRESSO, Mendelian randomization pleiotropy residual sum and outlier; NA, not applicable.

Figure 1: Flowchart for MR Analysis of COPD and T2DM





Figure 2: Forest Plot of the Main Results from the MR Analysis of COPD and T2DM


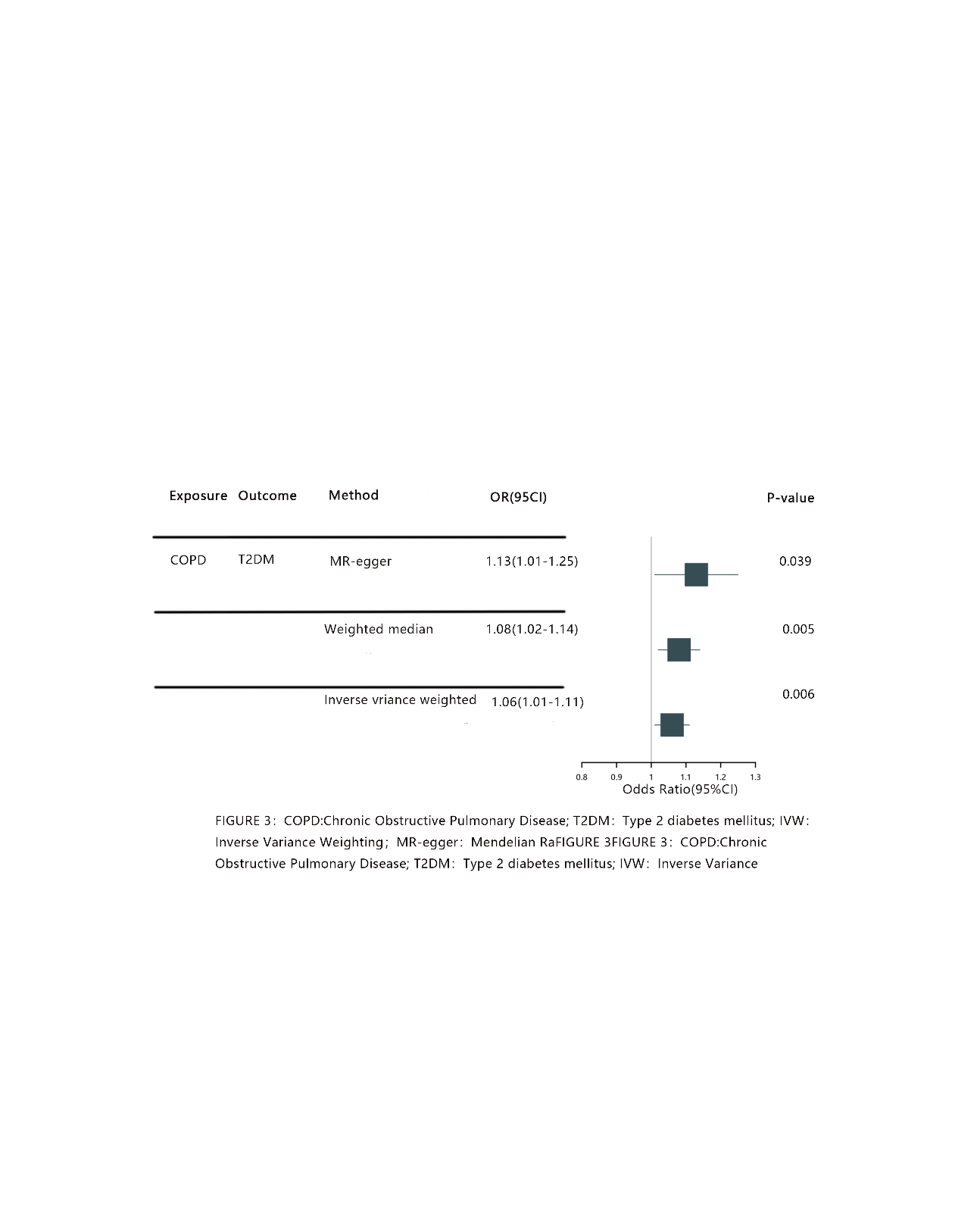


Figure 3: Scatter plot depicting the causal effect of COPD on the odds ratio for T2DM


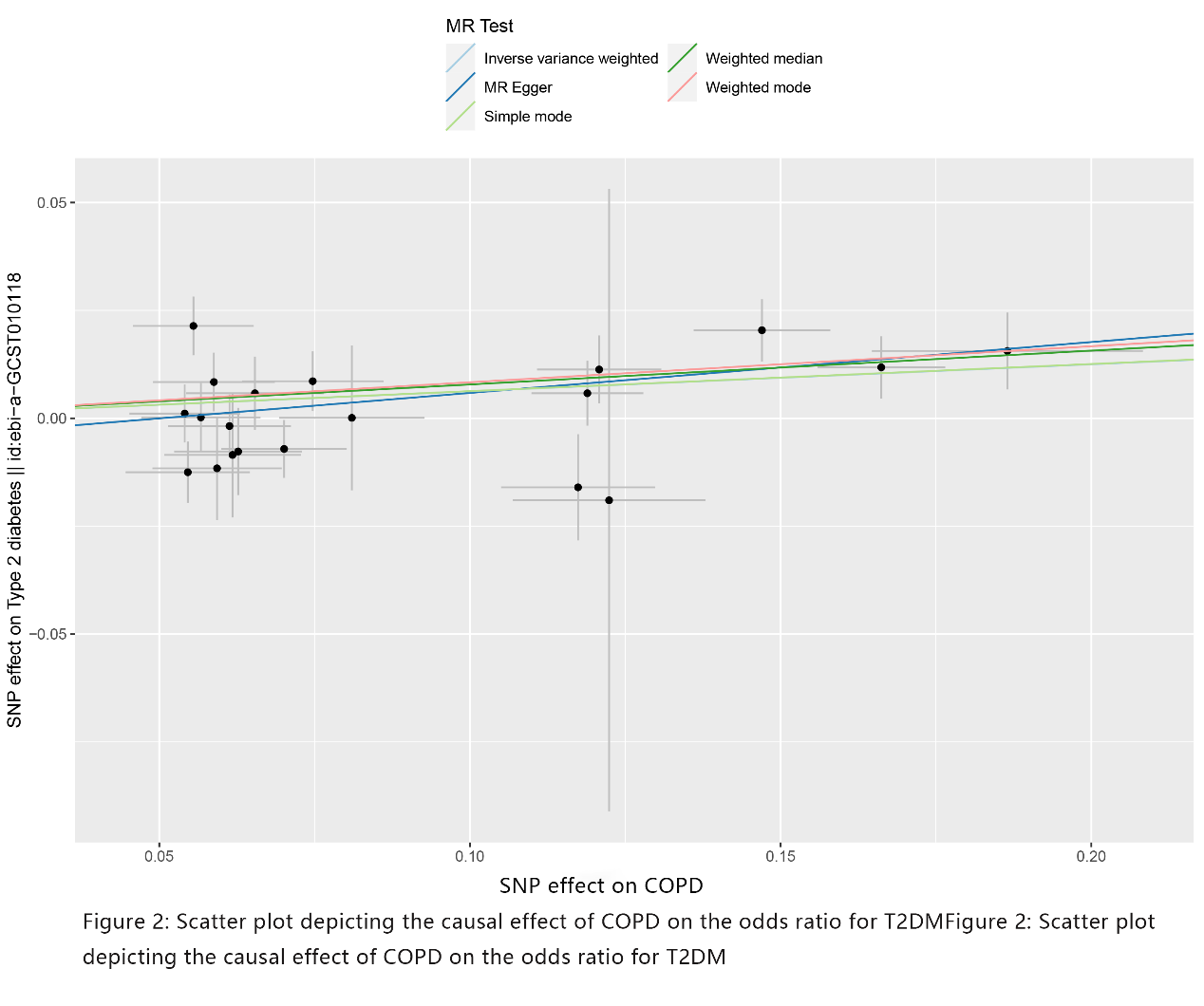


Figure S4 MR leave-one-out sensitivity analysis for COPD on T2DM


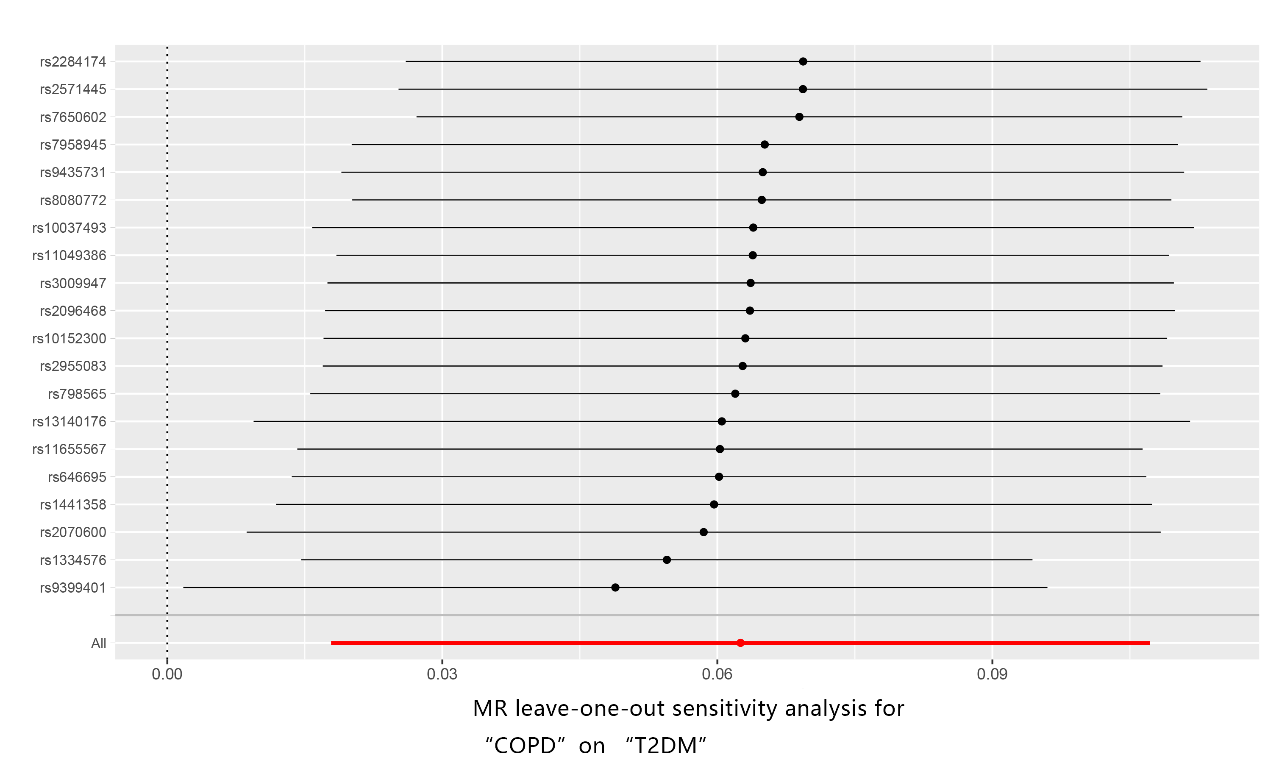


Figure S5 Funnel plot for COPD on T2DM


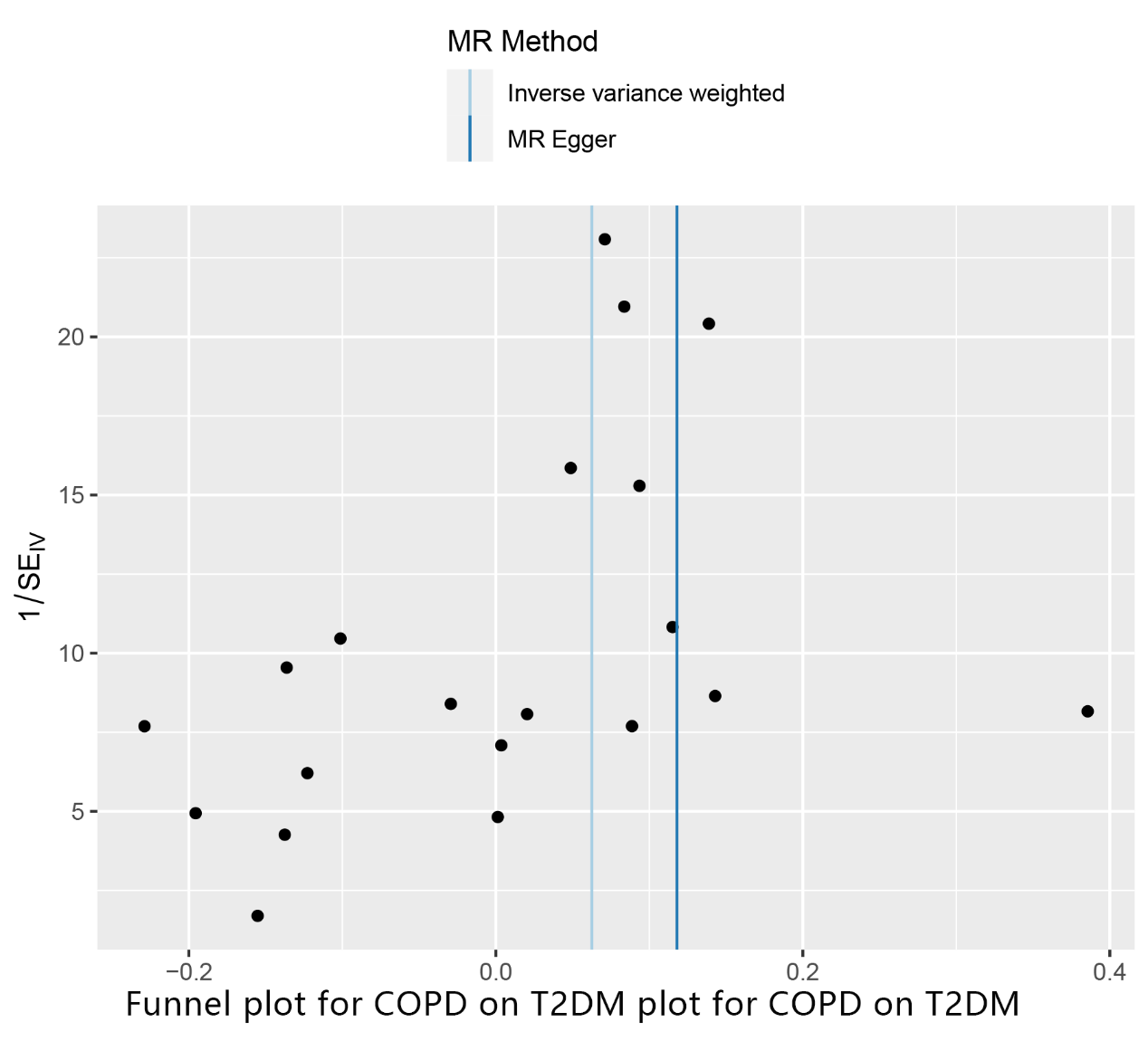


**
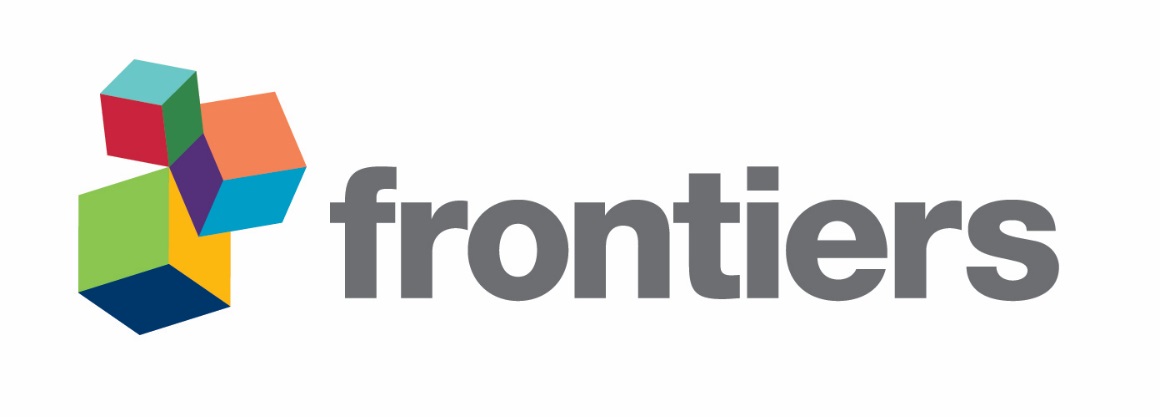
**
